# Vaccine equity in Africa: a rapid review of policy, legal and governance frameworks to improve research and development

**DOI:** 10.3389/frhs.2026.1678143

**Published:** 2026-08-04

**Authors:** John Ele-Ojo Ataguba, Grace Njeri Muriithi, Daniel Malik Achala, Elizabeth Naa Adukwei Adote, Chijioke Osinachi Nwosu, Senait Alemayehu Beshah, Elias Asfaw Zegeye, Fadima Inna Kamina Yaya Bocoum, Jemima Sumboh, Naomi Setshegetso, Nadia Yakhelef, William Muhwava

**Affiliations:** 1 African Health Economics and Policy Association (AfHEA), Accra, Ghana; 2 Department of Community Health Sciences, Max Rady College of Medicine, Rady Faculty of Health Sciences, University of Manitoba, Winnipeg, MB, Canada; 3 Partnership for Economic Policy (PEP), Nairobi, Kenya; 4 School of Health Systems and Public Health, University of Pretoria, Pretoria, South Africa; 5 Department of Economics and Finance, University of the Free State, Bloemfontein, South Africa; 6 Health System and Research Directorate, Ethiopian Public Health Institute, Addis Ababa, Ethiopia; 7 Division of Health Economics and Financing, Africa Centers for Disease Control and Prevention, Addis Ababa, Ethiopia; 8 Institut de Recherche en Sciences de la Santé, Ouagadougou, Ouagadougou, Burkina Faso; 9 University of York, Hull York Medical School, York, United Kingdom; 10 Department of Health Policy, Planning and Management, School of Public Health, University of Ghana, Accra, Ghana; 11 Department of Economics, University of Botswana, Gaborone, Botswana; 12 United Nations Economic Commission for Africa, Demographic and Social Statistics Section, African Centre for Statistics, Addis Ababa, Ethiopia

**Keywords:** Africa, COVID-19, equity, research and development, TRIPS, vaccine

## Abstract

**Background:**

Vaccines are instrumental in promoting preventive health and improving health outcomes globally. However, stark inequities in vaccine production, distribution, and access disproportionately burden low and middle-income countries (LMICs), which suffer significantly from these disparities. The COVID-19 pandemic further reinforced these existing inequities, prompting renewed debates on scaling up Africa's research and development (R&D) capacity as a proactive approach to enhance very limited domestic vaccine production and counter persistent inequities. However, there has been no consolidated review of the policy, legal, and governance frameworks that shape this capacity and their implications for vaccine equity. This rapid review asked which policy, legal, governance, and investment frameworks influence vaccine R&D and manufacturing capacity in African countries, and how they affect the continent's ability to secure equitable access to vaccines.

**Methods:**

We conducted a PRISMA guided rapid review of peer-reviewed and grey literature. Searches were conducted in Academic Search Premier, Business Source Premier, CINAHL via EBSCOhost, EconLit, Embase, and Ovid Medline via OVID Platform, as well as Google Scholar databases. Eligible documents reported on African vaccine R&D, manufacturing, or related policy, legal, governance, or investment frameworks. Grey literature was identified from the WHO, the World Bank, the Africa CDC, government organizations, private institutions, and policy think tanks. Data was extracted using a structured template and synthesized narratively into thematic areas that aligned with the study objectives.

**Findings:**

From 18,038 records, 68 publications met the inclusion criteria. We identified twelve active vaccine manufacturers in Africa and eleven additional initiatives in development, with the majority focusing on fill-and-finish operations and relatively few investing in upstream R&D and drug substance manufacturing. Persistent under-investment in vaccine R&D, fragmented and weak regulatory systems, and dependence on external financing constrain sustainable local manufacturing. Global and regional instruments, including the TRIPS Agreement, the Doha Declaration, ACT-A/COVAX, and the Pandemic Influenza Preparedness (PIP) Framework, create opportunities for technology transfer and impose intellectual property and supply-chain barriers that limit expansion of African vaccine R&D.

**Conclusion:**

Africa has the capacity to produce vaccines and related health products. However, this potential is undermined by structural under-investment in R&D, constrained access to technologies and inputs, and weak coordination of policy and regulatory frameworks. Strengthening domestic R&D financing, enhancing regional regulatory and policy harmonization, and proactively engaging in IP and trade negotiations, alongside strategic public-private and product development partnerships, are critical to achieving vaccine equity for African populations.

## 1 Introduction

Vaccines are unquestionably among the most cost-effective and equitable public health interventions (1), contributing to the decline in morbidity and mortality rates, the prevalence of some of the deadliest diseases, and the eradication of smallpox (2). Globally, immunization is reported to have averted 154 million deaths since 1974, including 146 million deaths among children below 5 years, out of which 101 million were infants below 1 year (3). It is further reported that an estimated 40% of the observed decline in global infant mortality, and 52% in the African region, is attributed to immunization (3). In 98 Low and Middle Income Countries (LMICs), about 37 million deaths, representing a 45% decline in deaths due to 10 vaccine-preventable diseases, have been averted (4). Also, it has been projected that from 2011 to 2030, immunization would avert the cost of illness of about $1,510.4 billion in 94 LMICs, and generate a benefit of $3,436.7 billion (4). Routine immunization, a key tenet of the World Health Organization Expanded Programme on Immunization, against diseases such as pneumonia, diarrhea, measles, and meningitis, has been instrumental in averting such deaths (4).

In Africa, over the years, routine vaccination has resulted in improved vaccine coverage rates, the eradication of polio, the introduction of new vaccines, and an improved investment landscape for research and development (R&D) capacity and infrastructure for vaccine production. Child coverage rates for the full series of three polio doses increased from 54% in 2000 to 73% in 2023, and yellow fever vaccination coverage also increased from 9% to 48% over the same period. Additionally, immunization for the full series of the two doses of measles increased from 5% in 2000 to 49% in 2023 (5). The success of these immunization programmes in LMICs and Africa is largely attributed to financial support and technical assistance from international donors and peculiar country context (4).

Despite significant progress and successes in vaccination programmes across the globe, there are stark inequities in the production of and access to these vaccines across countries and even within countries, with persistent global debates on how to ensure equitable access to vaccines. Africa's vaccine demand accounts for 25% of the global volume, with an estimated 1.5 billion doses needed annually, a figure projected to exceed 2.7 billion doses by 2040 (6, 7). Despite this, Africa's vaccine market accounts for just 4% of the global market, which is valued at 33 billion USD (6–8). It is estimated that only 1% of vaccines used in Africa are produced locally. Thus, most African countries are compelled to depend largely on imported vaccines to meet the needs of their national vaccination programmes (6–8). This underscores an unsustainable dependency on international aid and the region's susceptibility to the volatile dynamics of the global vaccine market (7). To reduce the risks of insufficient routine vaccination on public health and societal stability, safeguard against potential global health crises, and promote self-sufficient local healthcare economies, investing in Africa's domestic vaccine manufacturing capacity is crucial (7).

Global debates are emerging on improving Africa's capacity for vaccine R&D. The coronavirus pandemic in 2019 (COVID-19) triggered a re-emergence of such debates on scaling up immunization in Africa by improving its R&D capacity and prioritizing the domestic production of vaccines and medications. This is because, in the wake of the COVID-19 virus, barely a year after its emergence, vaccines were developed, tried, approved, distributed, and administered mostly in developed countries. This was an unprecedented achievement, since the development of vaccines involves a lengthy, cumbersome, and complex process, typically spanning between eight and fifteen years (9). The infectious and deadly nature of the COVID-19 virus incentivized global leaders, stakeholders, and public health experts to advocate for the fair and equitable distribution and access to vaccines under the broad mantra “no one is secure until everyone is secure” (10). Despite the significant feat of developing a COVID-19 vaccine and campaigns and pledges for global equity, numerous barriers to global vaccination persisted. Consequently, this resulted in vaccination inequities between countries and, to some extent, vaccine hesitancy (11). A study investigating COVID-19 vaccine hesitancy in Nigeria, for instance, reported that among the 56% of participants, only 10% were willing to receive the vaccine (11). Most of the reasons attributed to vaccine hesitancy included a lack of confidence in vaccines, fear of possible side effects, and limited access to vaccines (11).

Currently, only about 47% of the global population is either unvaccinated or only partially vaccinated, with accompanying stark disparities within countries, with the range of fully vaccination varying between 0% and 95% (12). As reported by Kreier (111), as of April 2021, about three-quarters of all COVID-19 doses had been administered in 10 countries, and only 2% had been administered in Africa. This implies a COVID-19 vaccination population coverage rate of just 1.4% of the entire African population (10). Whereas the inequitable distribution of vaccines, within and between countries, has impeded the progress towards equitable health systems, it has also brought to the forefront of discussions on vaccine equity the urgency for African countries, which over the years have disproportionately been disadvantaged in access to vaccines, to build up their research capacities for the domestic production of vaccines and medications.

Despite increasing attention to Africa's vaccine manufacturing agenda, most of the empirical evidence on Africa's vaccine manufacturing capacity is focused on individual countries, specific technologies, or broad political commitments, rather than providing a consolidated review of the policy, legal, and governance frameworks that underpin vaccine R&D and manufacturing across the continent. There is limited synthesis of how these frameworks intersect with global trade, intellectual property, and financing arrangements, and the resultant implications for vaccine equity. To address this gap and build on lessons from the COVID-19 pandemic, we conducted a rapid review of peer-reviewed and grey literature to map the policy, legal, governance, and investment frameworks that underpin vaccine R&D and manufacturing in Africa and to examine how these frameworks impact vaccine equity and global vaccine diplomacy. Although the acute phase of the COVID-19 pandemic has passed, decisions on vaccine R&D and manufacturing capacity in Africa (including investment, regulatory strengthening, and technology transfer governance) remain active and time-sensitive. A rapid review was therefore undertaken to provide a timely synthesis of the policy, legal, and governance frameworks and initiatives that shape this landscape, to inform ongoing planning and implementation. The specific objectives of this rapid review include:

1. To assess the existing policy, legal, governance, and investment frameworks supporting Africa's vaccine research and development capacity and production infrastructure, including the role of commercial, economic, and trade agreements in shaping vaccine development on the continent.
2. To examine stakeholder engagement models (including private sector participation) and innovative frameworks that contribute to equitable vaccine access and enhance Africa's position in global vaccine trade and diplomacy

The research question that guided the conduct of this study is:

Which policy, legal, governance, and investment frameworks influence vaccine R&D and manufacturing capacity in African countries, and how do they affect the continent's ability to secure equitable access to vaccines?

## 2 Methods

**Figure 1:**
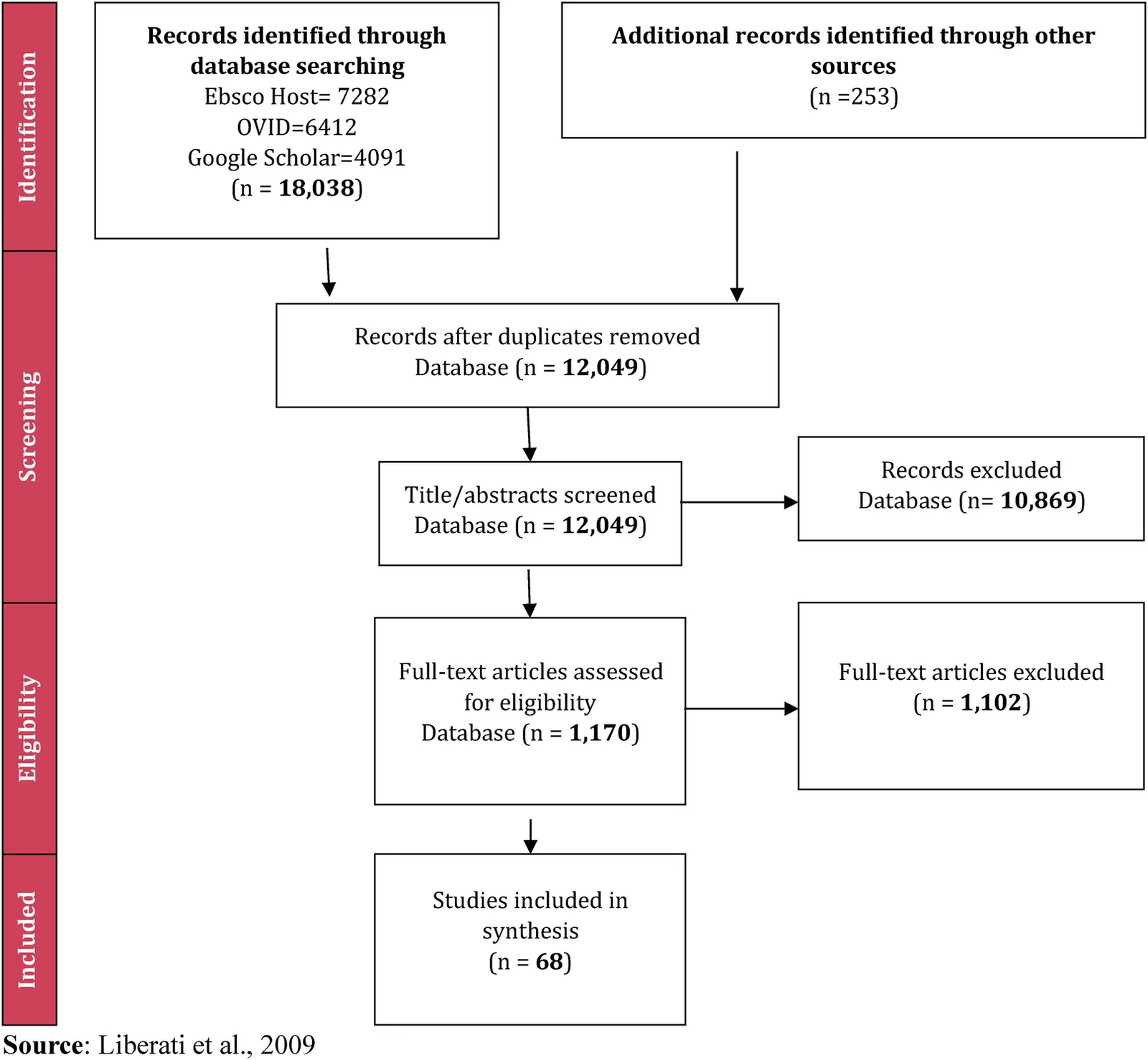
PRISMA flow diagram (112). License: Creative Commons Attribution License. Source: PRISMA 2009 flow diagram, published in PLoS Medicine. Use: Adapted. The PRISMA flow diagram/template was adapted and populated with the authors' own review screening numbers. The PLOS page lists the PRISMA article citation and states that it is distributed under a Creative Commons Attribution License, permitting reuse with proper credit. It also identifies Figure 1 as the flow of information through phases of a systematic review.

This rapid review was guided by the Preferred Reporting Items for Systematic Reviews and Meta-Analyses (PRISMA) guidelines (13). A rapid review is a method of knowledge synthesis designed to quickly provide preliminary insights into an urgent research question (14). It is an effective approach to generating an initial summary of the vaccine development landscape in Africa. The methods and results were reported using the PRISMA Flow Diagram (13). Figure 1 depicts the PRISMA flow diagram for the screening and selection of the reviewed articles.

### 2.1 Inclusion and exclusion criteria

The criteria for eligibility include: (1) Peer-reviewed articles and grey literature that were relevant to at least one of the objectives of the study, (2) Articles or reports should have been conducted in Africa or have information of an African country, and (3) Peer-reviewed articles and grey literature should be published in English. There were no time restrictions as part of the eligibility criteria.

The rapid review excluded: (1) Articles that were not relevant to at least one of the objectives of this study, (2) Articles or reports that did not have any information on any African country, and (3) Articles or reports published in languages other than English.

### 2.2 Search strategy and selection criteria

An initial thorough search was conducted in January 2023 and was not restricted by publication date, which reflected our intent to capture both pre- and post-COVID-19 policy and governance developments. To incorporate more recent evidence, the search was rerun using the same search strategy and eligibility criteria. Additional searches were conducted to identify literature published between February 2023 and May 2026. The databases searched included Academic Search Premier, Business Source Premier, CINAHL via EBSCOhost, Econlit, Embase, Ovid Medline via the OVID Platform, and Google Scholar to identify relevant studies for the analysis.

The initial search yielded 15,586 pieces of documented evidence, and the updated search yielded 2,452 articles, making a total of 18,038. All this evidence was uploaded into Covidence for screening and extraction. The duplicates were removed automatically when they were uploaded into Covidence. The analysis of this rapid review aligned with the PRISMA guidelines for highly transparent and complete reporting of reviews (15). The focus of the review was to understand the existing policy, legal, and governance frameworks that underpin Africa's capacity for R&D and how they contribute to global diplomacy for fair vaccine trade agreements that benefit the African population.

The search strategy for OVID Medline combined terms related to vaccines and research and development (example “vaccin*”. “research and development”, “vaccine production”) with terms related to policies, trade and governance (example “trade agreement*”, “TRIPS”, “industrial policy”, “Regulation”) and Africa-specific geographical terms (example “Africa*”, “sub-Saharan Africa”, and names of individual countries). We also used keywords and possible synonyms in the databases, such as vaccine, research and development, vaccine development, trade policies, vaccine production, policies, or agreements on vaccine production and distribution.

The titles and abstracts of all identified articles were screened. All the potentially relevant articles were retrieved in full and assessed for eligibility. The bibliographies of the identified articles were also screened to identify additional publications relevant to the study's objectives. Relevant grey literature on country- and/or regional-level policy documents and protocols, and reports on Africa's capacity for vaccine manufacturing, was accessed from the websites of organizations such as the WHO, the World Bank, government organizations, Africa CDC, private organizations, and policy think tanks. Most of the findings were retrieved from grey literature. Titles and abstracts were screened independently by two reviewers, with disagreements resolved through discussion, and full-text screening followed the same process. Grey literature sources from the WHO, World Bank, Africa CDC, and regional or national government websites were searched iteratively using targeted keywords and citation chasing. Figure 1 depicts the PRISMA flow diagram for the selection of publications for review.

### 2.3 Data extraction

A data extraction template was developed *a priori* based on the review objectives and piloted on three included documents to ensure clarity and consistency. During piloting, a small number of additional data items were identified as necessary to fully address the review objectives. These additions were agreed by the review team before full extraction was completed and then applied consistently across all included documents by re-checking previously extracted studies against the final template. Importantly, the review questions and eligibility criteria did not change; only the extraction fields were refined to improve completeness of capture. The following information was extracted: authors, title of paper or report, study objectives, the country in which the study was conducted, study design, study types, and type of vaccine under consideration. Additionally, detailed information was extracted on (1) the investment landscape for R&D capacity and infrastructure for vaccine production and development in Africa, (2) the reported policy instruments (ranging from commercial, economic, financial and trade agreements), impact on vaccine development in Africa, (3) interventions to Militate the Adverse Effects of these Agreements on Vaccine Development and Access in Africa, (4) existing Policy, Legal and Governance Frameworks to Improve Equitable Access to Vaccines and Capacity for Research and Development and Vaccine Production (5) frameworks to Boost Capacity for Innovation and Vaccine Development in Africa and (6) the Role of the Private Sector and other Key Stakeholders to Boost R&D for Vaccine Production in Africa.

### 2.4 Data synthesis and quality considerations

Data synthesis was conducted using Microsoft Excel. The data extracted from Covidence were organized into structured tables with columns for study characteristics and key findings. A narrative synthesis was adopted to synthesize the findings of the reviewed studies. The narrative approach was used because it enabled the combination of a wide range of different studies. The results from the reviewed studies were organized into themes aligned with the study's objectives. Then, the results of the reviewed studies were summarized using a narrative approach, and categorised into four thematic areas: (i) investment landscape for research and development capacity and infrastructure for vaccine production and development in Africa, (ii) the impact of commercial, economic, financial and trade agreements on vaccine development in Africa, (iii) existing policy, legal and governance frameworks to improve equitable access to vaccines and capacity for research and development and vaccine production, (iv) the role of the private sector and other key stakeholders to boost research and development for vaccine production in Africa.

In keeping with guidance for rapid reviews designed to inform timely policy debates, we did not undertake a formal risk-of-bias or quality appraisal for each included document. Instead, we prioritized documents from international organizations, regional institutions, and peer-reviewed journals.

## 3 Results

The search query yielded 18,038 records, of which 17,785 were identified through the key databases such as EBSCOhost, OVID, and Google Scholar. About 253 records were obtained from grey literature, such as the websites of the WHO, the World Bank, and Vaccine Manufacturing Companies in Africa, as well as through bibliography searches from the initial search. After removing duplicates (5,996), 12,049 titles and abstracts were screened. Of this number, 10,869 were excluded, and 1,170 were retained for full-text assessment based on the eligibility criteria. Only 68 met the inclusion criteria and were part of the research synthesis (see Figure 1).

### 3.1 Study characteristics

The key characteristics of the included studies or publications are shown in Table 1. The publications were predominantly focused on Africa (*n* = 30), with specific country-level publications from South Africa (*n* = 4), Nigeria (*n* = 5), Senegal (*n* = 3), Ethiopia (*n* = 2), Morocco (*n* = 2), Egypt (1), and Tunisia (*n* = 1). Approximately 20 publications were global in scope yet relevant to the African context, and 1 publication focused on developing countries. The included publications were mainly from grey sources; reports (*n* = 5), official websites of vaccine-producing companies or international organizations (*n* = 17), other relevant websites (*n* = 18), and then peer-reviewed articles (*n* = 12). The included publications ranged from literature reviews and program assessments to opinion articles and website information.

**Table 1:** Characteristics of included studies.

Author/institution	Year of publication	Title	Source type	Formality level	Country	Methods	URL/source link
Abiodun et al.	2021	Vaccine Manufacturing in Africa: What It Takes and Why It Matters	Report	Formal	Africa	Qualitative Review	https://www.tonyblairinstitute.org/reports/vaccine-manufacturing-africa
Africa CDC	2023	Our Work	Official Website	Semi-Formal	Africa	Program Assessment	https://africacdc.org/our-work/
African Vaccine Manufacturing Initiative	2019	Manufacturers	Report	Formal	Africa	Survey-based Review	https://www.avmi-africa.org/manufacturers/files/5017/manufacturers.html
Ahouidi et al.	2016	Malaria Vaccine Development: Focusing Field Erythrocyte Invasion Studies on Phenotypic Diversity	Journal Article	Formal	Africa	Case Study	doi: 10.1016/j.pt.2015.11.009
Aspen	2022	Aspen Pharmacare-Aspen 2022 Integrated Report	Report	Formal	Africa	Case Study	https://www.aspenpharma.com/
Asundi et al.	2021	Global COVID-19 vaccine inequity: The scope, the impact, and the challenges	Report	Formal	Global	Data Analysis	doi: 10.1016/j.chom.2021.06.007
Biovac	2023	Biovac	Journal Article	Informal	South Africa	Case Study	https://www.biovac.co.za/files/5013/www.biovac.co.za.html
Bregu et al.	2011	Accelerating vaccine development and deployment	Journal Article	Formal	Global	Case Study	doi: 10.1098/rstb.2011.0100
Brooks et al.	2012	Country planning for health interventions under development	Website	Informal	Global	Statistical Review	doi: 10.1093/heapol/czs039
BVNL	2017	BVNL-Company Profile	Website	Informal	Nigeria	Survey	https://www.biovaccinesnig.com/companyprofile.phpfiles/5023/companyprofile.html
Chang et al.	2023	The WTO Waiver on COVID-19 Vaccine Patents	Journal Article	Semi-Formal	Africa	Quantitative Analysis	https://www.uclalawreview.org/the-wto-waiver-on-covid-19-vaccine-patents/
Chaudhary and Chaudhary	2021	TRIPS waiver of COVID-19 vaccines	Website	Informal	Global	Descriptive Analysis	doi: 10.1111/jwip.12198
DCVMN	2023	Who we are & What we do – DCVMN	Journal Article	Formal	Africa	Qualitative Review	https://dcvmn.org/who-we-are-what-we-do/
Doua et al.	2025	Advancing Local Manufacturing Capacities for Vaccines within Africa	Journal Article	Formal	Africa	Narrative Review	10.1016/j.vaccine.2025.126829
EGY VAC	2023	EGY VEC	Report	Semi-Formal	Egypt	Cross-sectional Study	http://www.egyvac.vacsera.com/files/5019/www.egyvac.vacsera.com.html
Ethiopian Public Health Institute	2023	Ethiopian Public Health Institute – To be Center of Excellence in Public Health in Africa	Report	Formal	Ethiopia	Descriptive Analysis	https://ephi.gov.et/files/5015/ephi.gov.et.html
Ethiopian Public Health Institute	2021	Vaccine & Diagnostics Production – Ethiopian Public Health Institute	Journal Article	Formal	Ethiopia	Quantitative Study	https://ephi.gov.et/research/vaccine-diagnostics-production/
Fleck-Vidal et al.	2025	Landscape Analysis of the Vaccine Ecosystem in Africa	Report	Formal	Africa	Landscape Analysis	10.1016/S2214-109X(25)00314-6
Forman et al.	2023	Can We Move Beyond Vaccine Apartheid?	Journal Article	Formal	Global/Africa	Policy Analysis	10.1080/17441692.2023.2256822
Gennari et al.	2021	Africa needs vaccines. What would it take to make them here?	Website	Semi-Formal	Africa	Statistical Analysis	https://www.mckinsey.com/industries/life-sciences/our-insights/africa-needs-vaccines-what-would-it-take-to-make-them-here
Haakuria et al.	2025	Building Vaccine and Biotherapeutics Manufacturing Capacity in Africa	Journal Article	Formal	Africa	Narrative Review	doi: 10.1007/s44337-025-00313-w
Herder & Benavides	2024	WHO mRNA Technology Transfer Programme in South Africa	Journal Article	Formal	South Africa	Case Study	10.1371/journal.pgph.0003173
Innovative Biotech Nig LTD.	2022	Innovative Biotech Nig. Ltd	Report	Informal	Nigeria	Field Study	https://www.innovativebiotechng.com/about_us.htmfiles/5027/about_us.html
Institut Pasteur De Dakar	2019	Yellow fever vaccine production | Institut Pasteur de Dakar	Official Website	Semi-Formal	Senegal	Survey	http://www.pasteur.sn/en/yellow-fever-vaccine-productionfiles/5009/yellow-fever-vaccine-production.html
Institut Pasteur du Maroc	2017	Pharmaceutical Activities	Report	Semi-Formal	Morocco	Data Modeling	http://www.pasteur.ma/activitees_pharmaceutiques.php
Institut Pasteur Tunis	2023	Production	Official Website	Semi-Formal	Tunisia	Literature Review	https://www.un.org/africarenewal/magazine/december-2020/japan-senegal-vaccine-partnership
Kavanagh et al.	2021	Sharing Technology and Vaccine Doses to Address Global Vaccine Inequity and End the COVID-19 Pandemic	Report	Formal	Africa	Program Review	https://www.ncbi.nlm.nih.gov/pmc/articles/PMC5323145/
Kohler et al.	2022	Improving Access to COVID-19 Vaccines	Official Website	Formal	Africa	Data Modeling	doi: 10.1016/j.ijheh.2021.07.006
Kolawole et al.	2025	Challenges Implementing Technology Transfer as a Viable Pathway for Equitable Vaccine Production and Access	Journal Article	Formal	Africa	Case Study	doi: 10.1080/17441692.2025.2504698
Lamptey et al.	2022	COVID-19 vaccines development in Africa: a review of current situation and existing challenges of vaccine production	Report	Formal	Africa	Survey	https://www.oregonlive.com/health/2021/01/covid-19-vaccine-distribution-plans-in-africa-differ-by-country.html
Lardieri, A.	2021	Pfizer Partners With Biovac to Manufacture Coronavirus Vaccine in Africa	Report	Formal	Nigeria	Field Research	https://www.who.int/news-room/fact-sheets/detail/vaccine-equity
Makenga et al.	2022	Vaccine Production in Africa: A Feasible Business Model for Capacity Building and Sustainable New Vaccine Introduction	Website	Medium	Nigeria	Field Study	https://www.researchgate.net/publication/3035004
Manufacturing Africa	2023	A UK – Senegal Partnership Develops Covid 19 Rapid Test	Website	Semi-Formal	Global	Quantitative Study	https://www.mckinsey.com/business-functions/operations/our-insights/challenges-in-vaccine-production
Motari et al	2023	South Africa—blazing a trail for African biotechnology	Website	Medium	South Africa	Statistical Review	doi: 10.1016/j.ijheh.2021.08.006
Muanya, C.	2018	Nigeria's quest for local vaccines suffers setback	Official Website	Informal	Nigeria	Quantitative Review	https://www.who.int/information-centre/about-us
Mulumba et al.	2025	Decolonizing Global Health: Africa's Pursuit of Pharmaceutical Sovereignty	Journal Article	Formal	Africa	Conceptual Analysis	doi: 10.1186/s12913-025-13211-9
Mwila et al.	2025	Operationalizing African Self-Reliance in Vaccine Manufacturing	Journal Article	Formal	Africa	Policy Framework Analysis	doi: 10.1080/16549716.2025.2560209
Ndembi et al.	2024	Pandemic Agreement and African Health Security	Journal Article	Semi-Formal	Africa	Commentary	doi: 10.4102/jphia.v15i1.618
Nwaka et al.	2010	Developing ANDI: A Novel Approach to Health Product R&D in Africa	Journal Article	Medium	Africa	Statistical Analysis	https://www.borgenproject.org/vaccine-self-sufficiency-africa/
OBILADE, T.	2020	Made-in-Nigeria vaccines: Waiting for Godot!	Journal Article	Medium	Nigeria	Field Research	https://www.who.int/emergencies/diseases
Ogbodum et al.	2023	African Medicines Agency: How It Will Change the Landscape of Medicines in Africa	Journal Article	Formal	Africa	Policy Analysis	doi: 10.1002/puh2.96
Okesanya et al.	2026	Building Equitable and Sustainable Vaccine Ecosystems in Africa to Achieve Health Sovereignty and Universal Immunization by 2040	Journal Article	Formal	Africa	Narrative Review	doi: 10.1007/s44155-026-00364-z
Olu et al.	2025	Pandemic Accord Implementation in Africa	Journal Article	Semi-Formal	Africa	Policy Commentary	doi: 10.1186/s12992-025-01144-1
Ranjan, P.	2021	The Case for Waiving Intellectual Property Protection for Covid-19 Vaccines	Website	Informal	South Africa	Field Research	https://www.ox.ac.uk/academy/career-explorer-essays/covid-19
Roelf, W.	2019	Pfizer, Sanofi, to boost South African Biovac's vaccine output	Website	Semi-Formal	South Africa	Qualitative Research	https://www.mckinsey.com/industries/pharmaceuticals-and-medical-products/our-insights
Savoy and Leal	2021	Beyond COVAX: The Importance of Public-Private Partnerships for Covid-19 Vaccine Delivery to Developing Countries	Report	Medium	Africa	Cross-sectional Study	
Sensyo Pharmatech	2022	Home - Vaccine self-sufficiency and health independence - Sensyo Pharmatech	Official Website	Medium	Global	Qualitative Research	
Sibomana & Uwizeyimana	2025	Africa's Vaccines Manufacturing Revolution	Journal Article	Formal	Africa	Policy Analysis	doi: 10.24926/iip.v16i3.6592
Sithole et al.	2024	Comparison of Three Regional Medicines Regulatory Harmonisation Initiatives in Africa	Journal Article	Formal	Africa	Comparative Policy Analysis	doi: 10.34172/ijhpm.2024.8070
Spencer, D.	2020	Moroccan Pharma Companies to Know	Report	Medium	Morocco	Literature Review	
Sweitzer, S.	2021	Senegal's MADIBA Vaccine Facility	Website	Medium	Senegal	Field Study	
Sykes, A.	2002	TRIPS, Pharmaceuticals, Developing Countries, and the Doha “Solution.”	Website	Medium	Global	Quantitative Review	
Thambisetty et al.	2022	ADDRESSING VACCINE INEQUITY DURING THE COVID-19 PANDEMIC: THE TRIPS INTELLECTUAL PROPERTY WAIVER PROPOSAL AND BEYOND	Report	Medium	Global	Program Assessment	
Tsega	2025	Ensuring Vaccine Access Through Local Production	Journal Article	Formal	Africa	Legal and Policy Analysis	doi: 10.1093/jlb/lsaf020
United Nations Conference on Trade and Development	2020	Ten actions to boost low and middle-income countries’ productive capacity for medicines	Report	Medium	Global	Data Review	
Univercells	2023	Press Release: Institut Pasteur de Dakar to manufacture affordable Measles and Rubella vaccine	Website	Formal	Senegal	Descriptive Study	
UPS	2023	Our Company | About UPS	Report	Informal	Nigeria	Surveys	
Vawda	2021		Journal Article	Formal	Africa		
Wirsiy et al.	2025	Advancing Vaccine Manufacturing in Africa: A New Era for Immunisation Programmes towards Self-Sufficiency	Journal Article	Formal	Africa	Narrative Review	doi: 10.11604/pamj.supp.2025.51.1.46888
World Health Organization	2022	The ACT-Accelerator: Two years of impact	Official Website	Formal	Global	Program Evaluation and Impact Analysis	https://www.who.int/publications/m/item/the-act-accelerator–two-years-of-impact
World Health Organization	2022a	Impact and results	Official Website	Formal	Global	Program Results and Impact Reporting	https://www.who.int/initiatives/act-accelerator/impact-and-results
World Health Organization	2022b	The Pandemic Influenza Preparedness Framework (PIP)	Official Website	Formal	Global	Framework Description	https://www.who.int/multi-media/details/the-pip-framework
World Health Organization	2015	Regulatory collaboration: the African vaccine regulatory forum (AVAREF): a platform for collaboration in a public health emergency	Official Website	Formal	Africa	Policy analysis, collaboration framework	https://www.who.int/drugregulatory/news/WHO_drug_information_v29_2015.pdf
World Health Organization	2023a	COVID-19 Technology Access Pool (C-TAP)	Official Website	Formal	Global	Program overview, health initiative description	https://www.who.int/initiatives/covid-19-technology-access-pool
World Health Organization	2023b	Essential Programme on Immunization	Official Website	Formal	Global	Program Assessment	https://www.who.int/teams/immunization-vaccines-and-biologicals/essential-programme-on-immunization
World Trade Organization	1995	WTO | intellectual property (TRIPS) - agreement text - general provisions	Official Website	Formal	Global	Legal Text and Policy Framework	https://www.wto.org/english/docs_e/legal_e/27-trips_03_e.htm
Zipline	2023	Zipline - Instant Logistics	Official Website	Formal	Africa	Company overview, technology description	https://www.flyzipline.com/

The results of the rapid review are presented according to the two main objectives. First, we summarized the evidence on policy, legal, governance, and investment frameworks that shape Africa's vaccine R&D capacity and production infrastructure (Objective 1). Second, we presented findings related to stakeholder engagement, including private sector participation and innovative frameworks that influence equitable vaccine access and Africa's role in global vaccine trade and diplomacy (Objective 2).

### 3.2 Findings mapped to objective 1: policy, legal, governance, and investment frameworks

Objective 1 sought to assess the existing policy, legal, governance, and investment frameworks supporting Africa's vaccine R&D capacity and production infrastructure, including how commercial, economic, and trade agreements shape vaccine development. Three themes emerged: (i) the investment landscape for R&D and infrastructure, (ii) the impact of commercial, economic, financial, and trade agreements, and (iii) existing policy, legal, and governance frameworks.

#### 3.2.1 Investment landscape for research and development capacity and infrastructure for vaccine production and development in Africa

**Figure 2:**
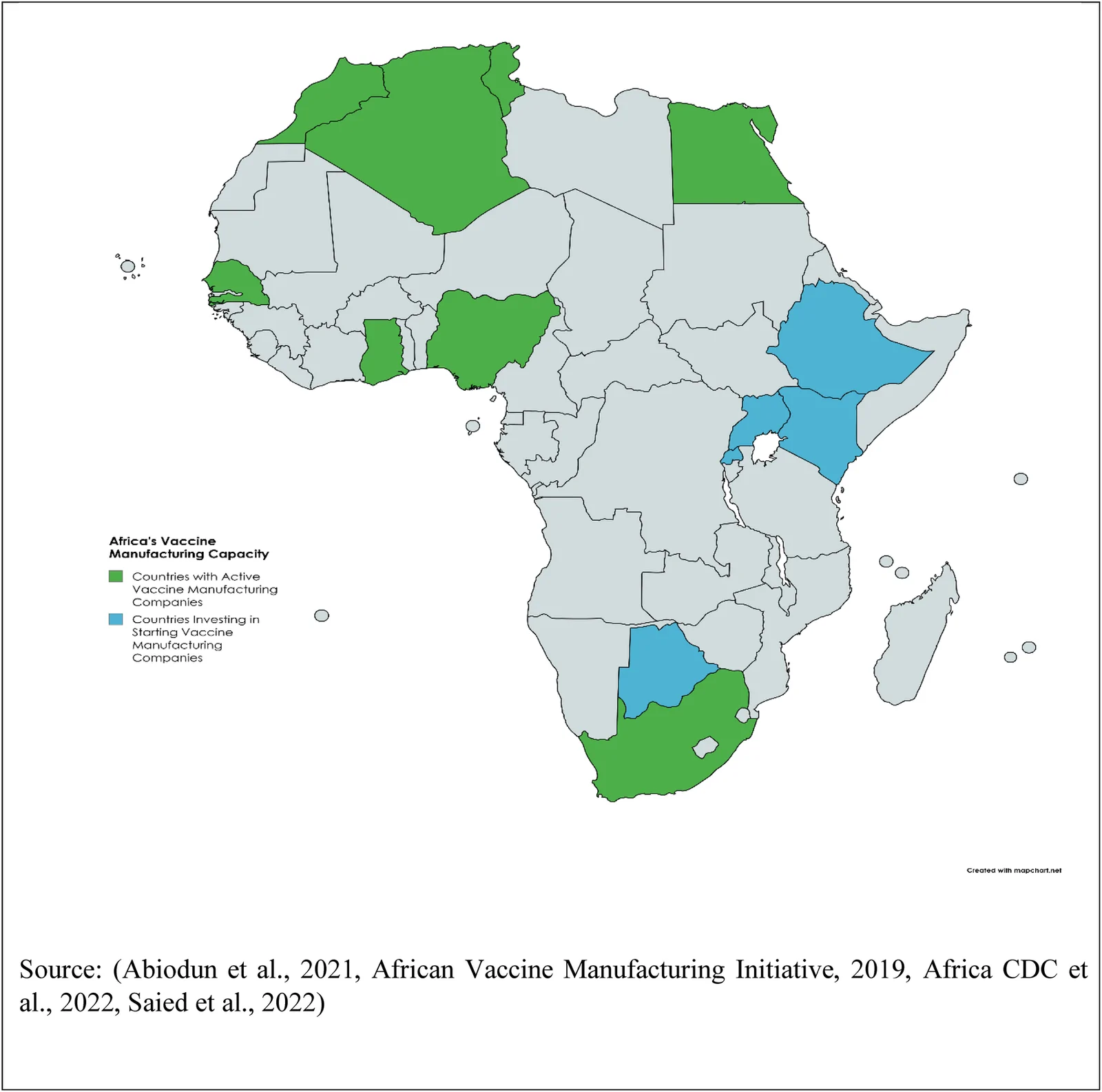
Africa's vaccine manufacturing capacity. Citation: Created using MapChart; data compiled/adapted from Abiodun et al. 2021; African Vaccine Manufacturing Initiative 2019; Africa CDC et al. 2022; and Saied et al. 2022. License: MapChart maps are licensed under Creative Commons Attribution-ShareAlike 4.0 International License, CC BY-SA 4.0. Source: MapChart map base; vaccine manufacturing capacity information compiled from the cited sources. Use: Adapted. The map was customized by the authors using MapChart and information from the cited sources; it was not reproduced as a complete figure from a single publication. MapChart's terms state that maps created with the website may be used, edited, and modified for personal, commercial, or publication use, provided MapChart is referenced; their maps are licensed under CC BY-SA 4.0.

The investment landscape for R&D and infrastructure in Africa is rapidly evolving, with both the public and private sectors playing increasingly active roles. Currently, Africa's vaccine market is estimated at $1.3 billion (91–110), and it is expected to increase to about $5.4 billion by 2030, all other things being equal (16). Africa is actively exploring more cost-effective ways of expanding its capacity to produce vaccines for its population. This is against the backdrop of rapid population growth and urbanization, gaps in immunization coverage, stringent trade policies, and stark inequities in the production and use of vaccines that the continent faces. However, due to inadequate investment capacity for research into the production of vaccines, the continent produces only 1% of its vaccine needs (17). There are currently 12 active vaccine manufacturing organizations in Africa with varying capacities, and most of these manufacturers are investing in fill and finish, with significant investment gaps and commitments to R&D, active pharmaceutical ingredients (API), and drug substance (see Table 2 and Figure 2 below), with an additional 11 companies still in the development stage.

**Table 2:** African companies across the vaccine manufacturing value chain Table 2, by country and stage of production (drug substance, fill-and-finish, packaging, research and development). This table illustrates the concentration of manufacturing capacity in a small number of countries and the dominance of fill-and-finish operations relative to upstream R&D and bulk production.

Continent	Country	Vaccine manufacturer	Year established	Vaccine	Manufacturing value chain stage
West Africa	Senegal	Institut Pasteur de Dakar^a^	1896	Yellow Fever	Manufacturing of drug substance Fill and finish Pack and label
	Nigeria	Biovaccines^b^		Intends to produce hepatitis B, yellow fever, tetanus, measles, Pentavalent vaccine, the recombinant hepatitis B vaccine, Tetanus with Diphtheria vaccine, Inactivate Polio Vaccine (IPV), the Pneumococcal Conjugate Vaccine (PCV), Human Papilloma Virus Vaccine (HPV) for cervical cancer, and Meningococcal vaccine	R&D Intend: Manufacturing of drug substance
		Innovative Biotech^a^	2005	HIV	R&D Pack and Label
	Ghana	DEK Vaccines Limited^b^	2022	Plan to Produce: Malaria, Pneumonia, Rotavirus, COVID-19 vaccines	Manufacturing of drug substance
		Atlantic Biotech^a^	2017	Sterile dosage forms including large volume Parenteral, eye/ear/nasal drops, small volume parenteral, biological products, vaccines and inhalational anaesthetic solutions	Manufacturing of drug substance
Southern Africa	South Africa	Biovac^a^	2003	BCG, Hepatitis B, Measles, Pneumococcal Conjugate, Hexavalent, Tetanus, Poliomyelitis, Tetanus Toxoid, Haemophilus Influenzae b infection	R&D Manufacturing of drug substance Fill and finish Pack and label
		Aspen^a^	1997	COVID-19	Fill and finish
	Botswana	Botswana Baylor Children's Clinic^b^	2026	Plan to Produce	Manufacturing of drug substance
East Africa	Ethiopia	Ethiopian Public Health Institute^b^	1995	Plan to Produce	Pack and label Import for sale
	Kenya	Afrigen^b^	2022	Plan to Produce	Manufacturing of drug substance
	Uganda	Dei BioPharma^b^	2022	Plan to Produce	Manufacturing of drug substance
	Rwanda	Rwanda Biomedical Center^b^	2023	Plan to Produce	Manufacturing of drug substance
North Africa	Egypt	Vacsera^a^	1881	BCG, DTP, Meningococcal Meningitis, Tetanus, Typhoid, Poliomyelitis, Cholera and Tuberculin	Fill and finish Pack and label
		Biogeneric Pharma^b^	2005	mRNA vaccines, therapeutic proteins and immunoglobulins (Plans to Produce)	R&D
		Vaccine Biotechnology City^b^		Plan to Produce: Measles, rubella and rotavirus vaccines	Manufacturing of drug substance
		Gennecs Group^b^	1957	Plan to Produce	Manufacturing of drug substance
		Eva Pharma^a^	1917	EgyVax (COVID-19 vaccine)	R&D Manufacturing of drug substance Fill and finish Pack and label
		Minapharma^a^	1958	Data unavailable	R&D Pack and Label
	Tunisia	Institut Pasteur Tunis^a^	1893	BCG, Rabies vaccine and bacterial vaccines for veterinary	Manufacturing of drug substance Fill and finish
	Algeria	Saidal^a^	1982	CoronaVac	Manufacturing of drug substance Import for sale
		Institut Pasteur d’Algerie^a^		Rabies	Manufacturing of drug substance Import for sale
	Morocco	Sensyo Pharmatech^b^	2022	Plan to Produce	Fill and Finish
		Institut Pasteur Du Maroc^b^	1929	Plan to Produce	Import for sale
		Marbio^a^	2021	Data unavailable	Fill and Finish
		Galenica^b^	1978	Sputnik (Plan to Produce COVID-19 (Plan to Produce)	Manufacturing of drug substance Fill and finish Pack and label

Source: (Abiodun et al., 2021, African Vaccine Manufacturing Initiative, 2019, Saied et al., 2022, Africa CDC et al., 2022).

a represent all active vaccine manufacturing companies in Africa and double asterisk.

b represent all other vaccine manufacturing companies in Africa at the development stages and planning to produce vaccines.

The findings emphasize that vaccine manufacturing extends beyond physical infrastructure and requires investments in research and development systems, clinical trial capacity, supply chains, workforce development, quality assurance systems, and innovation ecosystems (18–20). Persistent challenges include inadequate financing, limited technical expertise, fragmented markets, and uncertainty regarding long-term demand for locally manufactured vaccines (6, 21).

#### 3.2.2 The impact of commercial, economic, financial, and trade agreements on vaccine development in Africa

Two key Commercial, Economic, Financial, and Trade Agreements were identified to impact vaccine development in Africa. These were: (1) Trade-Related Aspects of Intellectual Property Rights (TRIPS) Agreement and (2) Access to COVID-19 Tools Accelerator (ACT-A). These instruments are discussed below.

##### 3.2.2.1 Trade-Related aspects of Intellectual Property Rights (TRIPS) and Its Impact on Vaccine Production in Africa

**TRIPS Agreement Overview**: Established in 1995 by the World Trade Organization (WTO), TRIPS aims to protect intellectual property rights globally. Article 7 of the TRIPS agreement highlights the objectives of the TRIPS agreement, describing in detail the protection and enforcement of intellectual property rights in the context of maximizing social and economic welfare, and balancing the rights and obligations of member states (22). The aim of Article 7 is to enhance knowledge sharing and technology transfer. However, it has been criticized for hindering the development of vaccines and therapeutics in developing countries by prioritizing private property protection over technology transfer and knowledge sharing (22, 23).

**Barriers to Vaccine Production**: TRIPS restricts generic manufacturing in Africa and other developing countries by granting patent holders exclusive rights for 20 years, preventing reverse-engineering of vaccines and medical supplies (24). This has resulted in high costs and limited access to essential medicines, as seen during the HIV/AIDS epidemic in South Africa, where the cost of antiretrovirals (ARVs) was unaffordable (25). A practical example is during the HIV/AIDS epidemic. The treatment of HIV/AIDS and containment of its spread were due to investments in R&D in developed countries, which subsequently resulted in containment in these countries (26). Antiretrovirals (ARVs) facilitated the treatment and containment of the diseases (26). However, in the late 1990s and early 2000s, there were deep inequalities in access to medicines and treatment for the HIV/AIDS epidemic, partly attributed to the TRIPS agreement (27). Then, the annual cost of advanced retroviral therapies in South Africa was approximately $12, 000 primarily due to the price offered by the pharmaceutical companies (25). The prices of the HIV drug fluconazole varied greatly across countries. For instance, the 150 mg of the HIV drug fluconazole costs $55 in India, relative to $687, $703 and $817 in Malaysia, Indonesia, and the Philippines, where the drug was patented (25). The procurement of medicines was expensive, and the procedure was cumbersome for most developing countries. Yet they could not reverse-engineer medications due to the patent protection these manufacturing companies enjoyed under the TRIPS agreement and had to rely on high-cost medicines and treatments. South Africa and most parts of Southern Africa were only able to contain the HIV epidemic after a Non-governmental Organization (NGO) sued the South African government over not permitting Cipla, an Indian Pharmaceutical company, to manufacture and sell cheaper generic medications. The cost of HIV treatment has reduced significantly to about 97% (26). This further introduced competition into the market for ARVs and significantly reduced the prices of patented ARVs.

**Doha Declaration**: To mitigate the adverse effects of the TRIPS agreement, the WTO introduced the Doha Declaration, allowing compulsory licensing for public health emergencies (26). With such cases, compulsory licensing is used to reduce the cost of medical supplies in the absence of voluntary licensing (26). However, challenges in access to affordable medical supplies, including COVID-19 vaccines, remain, as voluntary and compulsory licensing agreements often lack transparency and impose geographical restrictions that prevent export to developing countries, and only have a marginal impact on overall market supply (28).

**TRIPS Waiver Proposal**: South Africa and India have proposed a TRIPS waiver for COVID-19-related products, including vaccines, therapeutics, and diagnostics. This would allow local manufacturers to produce and export vaccines without legal constraints, facilitating technology transfer and enabling developing countries to manufacture their own vaccines (29). However, the waiver faces strong opposition from developed countries (29).

**mRNA Technology Transfer Programme**: The WHO mRNA Technology Transfer Programme in South Africa was identified in the literature as a novel model for sharing manufacturing knowledge and technical expertise across LMICs (30). Some studies reported that the programme has facilitated capacity building and knowledge exchange, but continues to face challenges relating to intellectual property restrictions, financing, commercial sustainability, and access to proprietary technologies (30, 31). Technology transfer, voluntary licensing agreements, collaborative manufacturing arrangements, and regional partnerships may be as important as intellectual property reform in strengthening vaccine production capacity in Africa (31–33). However, concerns remain regarding unequal access to technology, dependence on external partners, and limited bargaining power for African manufacturers within global pharmaceutical markets (34, 35).

##### 3.2.2.2 Impact of access to COVID-19 tools accelerator (ACT-A) and vaccine production in Africa

**ACT-A Overview**: The ACT-A was established by the WHO in April 2020. ACT-A is a partnership with governments, philanthropic and global health agencies, civil society, academia, and industry with the objective of accelerating the development, production, and equitable access to COVID-19 diagnostics, treatments, vaccines, and PPE. It is comprised of four pillars: diagnostics, treatment, vaccines, and health system strengthening, with a budget of US$38.1 billion for its full implementation (36).

**COVAX Facility**: The vaccine pillar, established by the COVAX mechanism and co-led by the WHO, CEPI, Gavi, and UNICEF, aims to provide equitable access to vaccines globally. By 2022, COVAX had delivered over 1.4 billion doses to 145 countries, including 92 low-income nations, through the Advance Market Commitment (AMC) (36).

**Support for African Manufacturing**: ACT-A has contributed to improving vaccine manufacturing capacity in Africa. In Senegal, the DIATROPIX venture supports affordable diagnostics, while the WHO mRNA vaccine hub aims to enable local production of mRNA vaccines in Africa, with Senegal among the six countries selected for this initiative (37). ACT-A through FIND and Unitaid, in collaboration with IPD Senegal, supports Senegal's DIATROPIX, a social venture to promote access to affordable and high-performance rapid diagnostics tests made in Africa, to expand COVID-19 Ag-RDTs capacity in Africa through technology transfer of two rapid tests for production at DIATROPIX, construction of a new facility to accommodate expanded manufacturing capacity (target of 50 million tests per year by 2023), and purchase of equipment to support manual and semi-automated manufacturing of new products (37). Also, the MADIBA initiative is supported by ACT-A (37). In February 2022, Senegal was selected in the first round of 6 countries that will receive the technology needed to produce mRNA vaccines in Africa through the WHO mRNA vaccine hub (37). In addition to supporting the procurement, delivery, and roll-out of vaccines, COVAX agencies and partners, including the ACT-A Facilitation Council, are helping to address key vaccine manufacturing bottlenecks to increase overall supply. For example, CEPI and the COVAX Manufacturing Task Force established an input-supply marketplace to facilitate the trade of supplies necessary to produce vaccines. WHO, with support from COVAX partners and the Medicines Patent Pool, established an mRNA technology transfer hub with multiple spokes worldwide to ensure diverse geographic production of vaccines (37).

Through the COVAX facility, about US$7 million was awarded to three self-test manufacturers to enhance the accessibility of COVID-19 Ag-RDT self-tests in LMICs (37). In addition, the ACT-A enhanced the engagement of public and private partners in African countries such as Kenya and Rwanda to pilot Ag-RDT professional use in pharmacy and occupational settings (37). The World Bank, via the ACT-A initiative, disbursed US$ 4 million to Senegal to strengthen its vaccination systems and general health system (37).

**Challenges in Vaccine Production**: Despite COVAX's success in vaccine distribution, ACT-A has struggled to build sustainable vaccine production capacity in Africa. COVAX has been unsuccessful in facilitating equitable access to vaccines by removing supply constraints and enhancing the domestic capacities of African manufacturers (23). The production of most of the widely recognized and used vaccines occurred in developed countries (27). Just a handful of voluntary licensing agreements targeted at increasing vaccine production capacity were established. For instance, J and J and Pfizer-BioNtech have accepted to partner with Aspen and Biovac, respectively, in South Africa in a limited “fill and finish” deal (27).

**Intellectual Property Barriers**: The TRIPS agreement has further constrained Africa's ability to scale up vaccine production. Issues such as patent restrictions and the dependency on raw materials from developed countries limit local manufacturing capacity (38). Another key barrier to COVAX is the shortage of raw materials and inputs for vaccine manufacturing in Africa. The COVID-19 vaccines that are intended to be manufactured in Africa with support from COVAX require an active ingredient produced in developed countries and shipped to these “finish and fill” facilities (38). However, the TRIPS agreement grants manufacturers control of such supplies. A typical example is the mRNA caps used in the manufacturing of the mRNA vaccine. These mRNA caps are produced by one specific company, which has patent rights over these caps and thus constrains the number of doses that can be produced, since manufacturers in Africa do not have the technical capacity to produce these mRNA caps (38).

**Technological Barriers**: Despite efforts to share technical knowledge (e.g., Moderna's temporary patent waiver), the lack of access to essential technologies (e.g., mRNA caps) has delayed vaccine production in Africa (38). Moderna's decision not to enforce its COVID-19 vaccine patents during the pandemic enabled Afrigen in South Africa to reverse-engineer the Moderna vaccine, and it is currently in the end stages of developing an mRNA vaccine comparable to the Moderna vaccine (27). Although the lack of shared knowledge from Moderna has delayed the Afrigen development process, it has shown that African manufacturing companies have the capacity to produce their own vaccines and medical supplies to meet the needs of the African population when technical knowledge is shared from the development countries through the implementation of such waivers of intellectual property.

**C-TAP Initiative**: The COVID-19 Technology Access Pool (C-TAP) was launched in May 2020 by the WHO, Costa Rica, and 40 other countries. C-TAP aimed to pool intellectual property and technical expertise to combat COVID-19. However, it faced significant challenges in garnering support from major manufacturers, which limited its effectiveness in facilitating technology transfer (23).

#### 3.2.3 Existing policy, legal and governance frameworks to improve equitable access to vaccines and capacity for research and development and vaccine production

There are a number of policy, legal, and governance frameworks implemented in Africa to improve equitable access to vaccines and to strengthen capacity for research and development and vaccine production. These include the Pandemic Influenza Preparedness (PIP) Framework, Ten Actions to Boost Low and Middle-Income Countries’ Productive Capacity for Medicines, The COVID-19 Tools Accelerator (ACT-A), The COVID-19 Technology Access Pool (C-TAP), The TRIPS waiver on COVID-19, The South African National Biotechnology Strategy of 2001, and the Essential Programme on Immunization (EPI). The frameworks are crosscutting, with some ensuring pandemic influenza surveillance, preparedness, and response systems, enhancing incentives for the biotechnology sector, enhancing faster, equitable, and affordable access to COVID-19 health products, as well as accelerating global collaboration to accelerate development, production, and equitable access to COVID-19 tests, treatments, vaccines, and PPE. Notable achievements from some of the policy frameworks include enhanced capacity for African manufacturers, resulting in the domestic production of key vaccines such as BCG, measles, and tetanus, amongst others (see Table 3 below for more details).

**Table 3:** Existing policy, legal, and governance frameworks to improve equitable access to vaccines and capacity for research and development and vaccine production.

Authors	General description of framework	Specific dimensions and criteria of framework	Achievements
World Health Organization (39)	The Pandemic Influenza Preparedness (PIP) Framework was created by the WHO in 2020 to improve global readiness and response to pandemic influenza.	- Focuses on sharing viruses with pandemic potential (like H5N1) and ensuring fair distribution of benefits. - Excludes seasonal influenza viruses and other non-influenza pathogens. - Countries are involved in an access and benefit-sharing system, contributing to vaccine development.	- WHO invested over USD 130 million into vaccine development capacity building. - The Global Influenza Surveillance and Response System (GISRS) includes over 150 laboratories, contributing over 1,200 PIP biological materials. - WHO secured 10% of global vaccine production capacity. - More than 90% of GISRS labs contributed to COVID-19 efforts.
United Nations Conference on Trade and Development (40)	A strategy to boost low and middle-income countries’ capacity for producing medicines and vaccines.	- Focus on investments in skills, infrastructure, and streamlined regulations for producing quality medicines. - Encourages partnerships and innovation, targeting sustainable development.	- Partnered with WHO, UNICEF, UNIDO, and others to build long-term capacity for LMICs. - Supports the ACT-A framework to facilitate equitable vaccine access.
World Health Organization (36)	The COVID-19 Tools Accelerator (ACT-A) aims to enhance global cooperation for equitable access to COVID-19 health products.	- Focuses on four pillars: diagnostics, therapeutics, vaccines, and health systems. - COVAX, a key part of ACT-A, ensures vaccine access and equitable distribution.	- COVAX delivered over 1.4 billion doses to 145 countries. - Over 80% of vaccines provided to low-income countries through COVAX. - Technology transfer hubs established in Africa, enabling local mRNA vaccine production.
World Health Organization (41)	C-TAP was launched to improve access to COVID-19 products by sharing intellectual property and data globally.	- Developers share COVID-19-related knowledge, data, and intellectual property through a global platform. - Supports agreements for technology transfer.	- The Medicines Patent Pool signed licenses with the Spanish National Research Council and NIH for COVID-19 diagnostics and therapeutics.
Chang (42)	The TRIPS waiver was proposed to temporarily relax intellectual property protections for COVID-19-related products to enhance global access.	- India and South Africa proposed a waiver in 2021 for patents, copyrights, and industrial designs related to COVID-19. - The waiver aimed to boost access in developing countries by allowing local production and reverse-engineering.	- The waiver has not yet been fully accepted but is expected to improve technology transfer and enable vaccine production in developing countries.
Motari et al. (43)	South Africa's National Biotechnology Strategy, implemented in 2001, aimed at promoting biotechnology innovation and industry growth.	- Focused on creating incentives for the biotechnology sector, including health and agricultural biotechnology. - Fostered innovation centers like the Cape Town Consortium and Biopad, which focused on research and development.	- Several innovation centers were established, and the government-run innovation fund supported technology transfer and incubation for small enterprises.
World Health Organization (44)	The Essential Programme on Immunization (EPI) ensures universal access to life-saving vaccines, starting with children.	- Established in 1974, the EPI focuses on ensuring vaccines are accessible to all children in every country. - Focuses on strengthening vaccine delivery systems and capacity building for vaccine production in African countries.	- National immunization programs are in place in all countries. - Significant improvements in immunization coverage in Africa, approaching 100% in some regions. - Supported domestic vaccine manufacturing, including BCG, measles, and polio vaccines.

### 3.3 Findings mapped to objective 2: policy, legal, governance, and investment frameworks

Objective 2 focused on stakeholder engagement models, including private sector participation and innovative frameworks that contribute to equitable vaccine access and strengthen Africa's position in global vaccine trade and diplomacy. Two key themes were identified: (i) frameworks to boost capacity for innovation and vaccine development, and (ii) the role of the private sector and other key stakeholders in expanding R&D and manufacturing capacity.

#### 3.3.1 Frameworks to boost capacity for innovation and vaccine development in Africa

In addition to the policy, legal, and governance frameworks in Africa aimed at improving equitable access to vaccines and R&D capacity, a few frameworks have been recommended to strengthen innovation and vaccine development capacity. Although some of these frameworks are specifically focused on African manufacturers, others are generic and targeted at improving the vaccine development processes.

##### 3.3.1.1 Policy and governance frameworks

Policy and governance frameworks were consistently identified as critical determinants of vaccine manufacturing capacity and vaccine equity in Africa. Studies highlighted the importance of political commitment, institutional coordination, industrial policy, and regional cooperation in creating an enabling environment for vaccine production and innovation. Governance initiatives led by the African Union, Africa CDC, and Partnerships for African Vaccine Manufacturing were frequently cited as important mechanisms for strengthening manufacturing capacity and reducing dependence on imported vaccines (21, 45, 46).

The African Union's objective of producing 60% of vaccines used on the continent by 2040 emerged as a central policy goal underpinning many manufacturing initiatives (21, 47). However, the successful implementation of this goal requires coordinated governance structures that align public health priorities, industrial development strategies, regulatory systems, procurement mechanisms, and investment policies.

The African Medicines Agency (AMA) was identified as a significant institutional development with the potential to strengthen regulatory governance, facilitate market access, improve coordination among national regulatory authorities, and support the growth of local pharmaceutical and vaccine manufacturing industries (48, 49). Regional regulatory harmonization initiatives were similarly recognized as important mechanisms to improve regulatory efficiency and reduce barriers to product registration across African markets.

Another key framework identified was the Bregu et al. Framework for Accelerating Vaccine Development and Deployment. Bregu et al. recommended four key steps to accelerate vaccine development and deployment. These are (1) setting vaccine discovery and development priorities, (2) implementing these vaccine discovery and development priorities, (3) establishing regulatory reform, and (4) increasing financial and public support for immunization programs (50). The framework emphasizes innovation in vaccine technologies, strengthening manufacturing and clinical testing infrastructure, multi-stakeholder financing, regulatory efficiency, and improved coordination among governments, industry, and global health organizations. Collectively, these components aim to accelerate the translation of vaccine research into licensed, accessible vaccine products (50).

The Africa Regional Guide to a Malaria Vaccine Decision-Making Framework was identified as another key framework to boost capacity for innovation and vaccine development in Africa. It was developed for use with any upcoming malaria vaccine (51). The framework is built on earlier work by the Malaria Vaccine Technology Roadmap to highlight the decisions the government would be required to make regarding the processes and data necessary for the introduction of a malaria vaccine (51). The decision-making framework was developed by African scientists, governments, and other key stakeholders in vaccine development. The framework is summarized in three key parts: pre-licensure, licensure, and post-licensure. Each stage of vaccine development requires different processes at the international and national levels, including the establishment of technical working groups; assessment and strengthening of regulatory, ethics, and data management processes; national expert group or technical working group recommendations; monitoring vaccine performance and safety; and vaccine implementation. The data required for policy development and implementation range from malaria disease burden and malaria vaccine impact to economic and financial issues, malaria vaccine efficacy, quality, and safety, programmatic considerations, and the socio-cultural environment (51).

##### 3.3.1.2 Legal, trade, and intellectual property frameworks

Legal, trade, and intellectual property frameworks emerged as important determinants of vaccine manufacturing capacity and access. Studies have highlighted the influence of patent protections, trade agreements, licensing arrangements, and intellectual property regulations on African manufacturers’ ability to acquire technologies and expand local production capacity (33, 34).

The TRIPS Agreement continued to feature prominently in discussions concerning equitable access to vaccine technologies. Evidence suggested that intellectual property protections may constrain local manufacturing where access to proprietary technologies, technical know-how, and production processes remains restricted. At the same time, studies emphasized that intellectual property reform alone is unlikely to resolve manufacturing challenges without complementary investments in infrastructure, workforce development, financing, and technology transfer systems (34, 35).

Technology transfer arrangements were frequently identified as practical mechanisms for expanding manufacturing capacity. Studies examining the World Health Organization's mRNA Technology Transfer Programme in South Africa highlighted both the opportunities and challenges associated with transferring manufacturing knowledge, technical expertise, and production capabilities to LMICs (30, 31). While technology transfer initiatives have strengthened local capabilities, barriers relating to intellectual property, sustainability, financing, and access to proprietary technologies remain significant concerns as discussed above.

##### 3.3.1.3 Financing and investment frameworks

Financing and investment frameworks were consistently identified as essential for the development of sustainable vaccine manufacturing ecosystems. Studies highlighted substantial funding gaps affecting vaccine research and development, manufacturing infrastructure, workforce development, and innovation systems across Africa (18). Evidence demonstrated increasing interest in financing mechanisms designed to stimulate local production and reduce investment risks. These included advance market commitments, pooled procurement arrangements, blended financing models, development finance initiatives, and the African Vaccine Manufacturing Accelerator (AVMA) (18, 21). Such mechanisms were viewed as important tools for creating predictable demand and improving the commercial viability of locally manufactured vaccines.

Several studies emphasized that sustainable manufacturing requires long-term investments extending beyond production facilities to include research institutions, supply chains, quality assurance systems, workforce development, and clinical trial infrastructure (18, 20). Concerns regarding dependence on donor financing, competition from established global manufacturers, and uncertainty in future procurement markets were identified as continuing challenges to investment sustainability.

Another key financing and investment framework identified was the Feasible Business Model for Capacity Building and Sustainable New Vaccine Introduction in Africa, developed to explore ways to enhance vaccine production across the continent. It was developed based on a literature review and a survey of key vaccine manufacturing stakeholders, such as local officials of sub-Saharan African government institutions, NRS officials of sub-Saharan African countries and non-African developing countries with vaccine manufacturers, members of Developing Countries Vaccine Manufacturers (DCVMs), Global vaccine manufacturers/ multinational companies (MNC), WHO-NRA capacity building officials, and other global vaccine stakeholders (51). The Feasible Business Model for Capacity Building and Sustainable New Vaccine Introduction in Africa was developed to assess pathways to strengthen vaccine manufacturing capacity across the continent. The model evaluates alternative production scenarios, investment requirements, regulatory capacity needs, and manufacturing sustainability considerations. It emphasizes the importance of long-term financing, manufacturing infrastructure, regulatory strengthening, and strategic planning to support sustainable local vaccine production and facilitate the introduction of new vaccines in African markets.

##### 3.3.1.4 Public–private partnerships and stakeholder engagement frameworks

PPPs and stakeholder engagement frameworks were identified as important enablers of vaccine manufacturing and vaccine equity. Studies consistently emphasized the need for collaboration among governments, manufacturers, academic institutions, international organizations, development partners, philanthropic organizations, and private-sector actors to support the development of manufacturing capacity (6, 20). Examples of multi-stakeholder collaboration included the WHO mRNA Technology Transfer Programme, initiatives coordinated through PAVM, manufacturing partnerships supported by Africa CDC, and collaborative arrangements involving Gavi, CEPI, and vaccine manufacturers (30). These initiatives contributed to workforce development, technology transfer, infrastructure investment, manufacturing readiness, and regulatory strengthening. The literature suggests that stakeholder engagement plays a critical role in aligning financing mechanisms, procurement strategies, industrial policies, and regulatory systems. Several studies have concluded that vaccine manufacturing capacity is most effectively strengthened through coordinated, ecosystem-wide approaches that integrate public and private actors, rather than through isolated interventions focused solely on manufacturing facilities (19, 20).

#### 3.3.2 The role of the private sector and other Key stakeholders to boost research and development for vaccine production in Africa

Several key stakeholders are instrumental in boosting R&D for vaccine production in Africa. These include African Vaccine Manufacturing Initiative (AVMI), West African Merozoite Invasion Network (WAMIN), the African vaccine regulatory forum (AVAREF), African Network for Drugs and Diagnostics Innovations (ANDI), Developing Countries Vaccine Manufacturers Network (DCVMN), Zipline and UPS Foundation in Ghana, Africa Centres for Disease Control and Prevention (Africa CDC), International Aids Vaccine Initiative (IAVI) and, various research institutes or centers in Africa such as the Ifakara Health Institute, Noguchi Memorial Institute, KEMRI-Wellcome Trust, as well as South African Medical Research Council (SAMRC) and various universities in Africa. The focus for these key stakeholders is discussed below:

1. African Vaccine Manufacturing Initiative (AVMI): AVMI is responsible for coordinating efforts of African vaccine manufacturers and other interested stakeholders to enhance the manufacturing of vaccines in Africa. It currently comprises four working groups responsible for advocacy, communications, vaccines and technology, and funding and finance. The AVMI has conducted assessments of the vaccine manufacturing capacity and procurement mechanism for establishing sustainable vaccine manufacturing capacity in Africa (52).
2. The West African Merozoite Invasion Network (WAMIN) was formed as part of the malaria vaccine development project to expand training in *Plasmodium* phenotyping and perform large-scale field phenotyping studies to prioritize blood stage vaccine candidates in Africa. WAMIN is a small group of scientists who are actively involved in erythrocyte invasion studies in West Africa, and have committed to working together to standardize protocols, train and certify young scientists, and share reagents and data (53).
3. The African vaccine regulatory forum (AVAREF) is a regional regulatory network founded by the WHO in 2006 that is made up of a network of national regulatory authorities (NRAs) and ethics committees of the countries in the WHO African Region (World Health Organization, 2015). It currently has 23 members. AVAREF seeks to provide technical support to NRAs in regulatory decision-making. It serves as a source of information on vaccine candidates and clinical trial timelines, and facilitates communication and collaboration between African NRAs and ethics committees. AVAREF is also instrumental in bringing expertise and recommendations of regulators from developed countries (54).
4. The African Network for Drugs and Diagnostics Innovations (ANDI): ANDI has the objective of partnering, funding, and coordinating research by setting up collaborative project networks and partnerships, building capacity and support for infrastructure development (Nwaka et al., 2010). It is also responsible for advocacy for investments in research and development and for building domestic intellectual property expertise. The research scope of ANDI includes drugs, diagnostics, vaccines, and medical devices. ANDI also advocates for the downstream research for health systems strengthening (55). ANDI comprises partners such as the WHO, the WHO African Regional Office, the WHO Eastern Mediterranean Regional Office, the African Development Bank, the European Union, and other national African institutions. ANDI is focused on complementing external research and development efforts and promoting product development partnerships (55).
5. Developing Countries Vaccine Manufacturers Network (DCVMN): DCVMN is made up of at least 40 vaccine manufacturers from 15 developing countries (South Africa is the only African country) (DCVMN, 2023). The DVCMN is involved in innovation, research, development, manufacturing, and supply of high-quality vaccines to 170 countries. It helps strengthen members’ capacity through advocacy and professional training programs. It also acts as a liaison to foster partnerships and funding, and encourages technology transfer initiatives (56).
6. Zipline and UPS Foundation in Ghana: Zipline is a private company leveraging expertise in robotics. Zipline designs, manufactures, and operates the world's largest automated delivery system (57). UPS Foundation is also a private package delivery company (58). In 2021, Zipline and the UPS Foundation partnered with the government of Ghana to distribute COVID-19 vaccines and PPE via the medical drone delivery system. This was to ensure the timely distribution of the COVID-19 vaccines and other medical supplies (59).
7. Africa Centers for Disease Control and Prevention (Africa CDC): The Africa CDC is responsible for strengthening the capacity and capability of Africa's public health institutions and detecting and responding to disease threats and outbreaks based on surveillance in a timely manner (Africa CDC, 2023). It is made up of six strategic pillars: strengthen integrated health systems to prevent and control high-burden diseases; build up proactive surveillance, intelligence, gathering and early warning systems; ensure robust emergency preparedness and response capabilities for public health emergencies; strengthen national public health institutes; expand clinical and public health laboratory systems and networks and expand health product and technology innovation and manufacturing (60).
8. International AIDS Vaccine Initiative (IAVI): IAVI seeks to develop an HIV vaccine through its clinical research activities in East Africa. It supports scientific excellence, good clinical practice, and has invested in infrastructure and laboratories at clinical research centers in East Africa (61).

### 3.4 Cross-Cutting synthesis of thematic findings

Taken together, the four thematic areas highlight how financing and investment, trade and IP agreements, policy and governance frameworks, and stakeholder roles interact to shape Africa's vaccine R&D and manufacturing landscape. Underinvestment in R&D and infrastructure limits African manufacturers’ ability to move beyond fill-and-finish operations, while global and regional trade and IP regimes constrain access to technologies and critical inputs. Policy, legal, and governance frameworks create important opportunities through instruments such as the PIP Framework, TRIPS flexibilities, ACT-A, and C-TAP, but their impact is mediated by the extent to which they are translated into actionable, adequately financed national strategies. Finally, the frameworks and stakeholder models described in Sections 3.3.1 and 3.3.2 illustrate that public-private and product development partnerships, regulatory collaboration platforms, and regional networks can help bridge these gaps but remain nascent and unevenly distributed across the continent. This interconnectedness underscores the need for coordinated interventions across all four pillars to achieve vaccine equity in Africa. The main findings of the included documents across the four thematic pillars are highlighted in Table 4**.**

**Table 4:** Summary of Key findings by thematic pillar.

Pillar	Key issues identified	Illustrative examples (Africa)	Implications for vaccine equity and R&D
Governance and regional coordination	Fragmented regional/national strategies; limited coordination of manufacturing initiatives; variable engagement with Africa CDC and AU platforms	Over-reliance on COVAX and ACT-A; uneven participation in regional surveillance and regulatory initiatives; limited cross-country partnerships	Weak bargaining power in global markets; duplication of efforts; slow translation of continental visions into national action
Legal and IP frameworks	TRIPS-related constraints; limited use of flexibilities; uncertainty around voluntary licensing; uneven domestic IP law reform	TRIPS waiver debates; constraints on access to inputs like mRNA caps; limited uptake of mechanisms such as C-TAP	Barriers to scaling up local production; dependence on foreign patent holders; constrained technology transfer
Financing and investment	Heavy reliance on external donors; underinvestment in R&D; limited domestic industrial financing mechanisms	Public–private partnerships focused on fill-and-finish; nascent investment in full value-chain capacity	Vulnerability to external funding cycles; difficulty sustaining long-term capacity building
Regulatory capacity and infrastructure	Fragmented regulatory standards; limited capacity of national regulatory authorities; uneven progress toward harmonisation	Variable adoption of AU and WHO regulatory initiatives; limited regional joint reviews and reliance mechanisms	Slow approval of new products; obstacles to regional manufacturing hubs serving multiple markets

## 4 Discussion

The evidence shows Africa's current investment landscape in R&D capacity and infrastructure for vaccine development, as well as the prevailing structures that impede its ability to expand vaccine manufacturing capacity. Although some countries have made significant investments in vaccine development and infrastructure capacity, others are lagging behind, with little or no investment in R&D capacity or vaccine development. The distribution of manufacturing capacity and policy initiatives across the continent is highly uneven. As shown in Tables 1, 2, a small number of countries, particularly South Africa, Senegal, Egypt, Morocco, Nigeria, and Tunisia, host most of the existing and planned manufacturing facilities and appear most frequently in policy and program documents. By contrast, many countries in Central and parts of West and East Africa have little or no documented vaccine manufacturing activity and rely almost entirely on imports and global mechanisms such as COVAX. This intra-continental disparity raises important equity questions within Africa itself and suggests that regional strategies will need to deliberately support countries with limited fiscal space, industrial bases, or regulatory capacity to benefit from emerging manufacturing hubs.

Currently, it is reported that countries in Sub-Saharan Africa typically contribute about 0.4% of their Gross Domestic Product (GDP) to scientific research, whereas countries in Europe, Asia, and North America contribute about 27%, 31%, and 37%, respectively, to R&D (62). Africa accounted for 1.1% of global R&D investment in 2016 (63). However, there were vast disparities within the continent, with South Africa, Egypt, and Nigeria accounting for 65.7% of R&D spending (63). In most developed countries, the largest funding source for R&D is the private sector. However, in Africa, R&D is primarily funded by the public sector and international funding (63). Insufficient funding and investment in R&D for vaccine production limit Africa's vaccine manufacturing capacity, resulting in the production of only 1% of its vaccine needs (17).

Despite these challenges, there have been several notable developments in Africa's vaccine R&D landscape. A recent Africa CDC African vaccine manufacturing landscape analysis reported the presence of ten commercial scale vaccine manufacturing facilities in Africa (Africa CDC, 2023) South Africa, with its vaccine manufacturing companies, Biovac and Aspen, take frontline positions in R&D and vaccine development in the continent, and have a comparative advantage in developing public private partnerships for vaccine development since South Africa is dominated by the private sector and demonstrated its ability to succeed in public private partnerships (64). Senegal, through the Institut Pasteur de Dakar, the only company currently producing vaccines in West Africa, has a comparative advantage in developing Yellow Fever vaccines in Africa. This is because it is the only African company, and one of the four global manufacturers, pre-qualified by the WHO for the manufacturing of the Yellow Fever vaccine (65). Usually, the process of obtaining certification for vaccine production is cumbersome and time-consuming, requiring compliance with stringent good manufacturing practice regulations.

It is evident from the findings that, despite the continent having at least seven vaccine manufacturing companies with varying capacities, most are at the fill-and-finish and drug substance manufacturing stages. Most vaccines are thus fully manufactured in parent companies, mostly in developed countries. Although it facilitates technology transfer from developed countries to African countries, such a system also provides developed countries with a home advantage and better access to vaccines than most African countries. These developed countries tend to prioritize national access over global equity, as was witnessed during the COVID-19 pandemic. In the event of an outbreak, epidemic, or pandemic, as witnessed during the COVID-19 pandemic, African countries are disproportionately burdened by low vaccination rates and inadequate medical supplies or products to contain the spread of the disease. Hence, the inequitable distribution of manufacturing capacities disproportionately affecting Africa inevitably results in the inequitable distribution of vaccines (17). A typical example was shown by Abiodun et al., who further revealed the stark inequitable distribution of COVID-19, where out of the total 8.6 billion COVID-19 doses that were secured by mid-March 2021, high and upper-middle-income countries had purchased about 6 billion doses, whereas LMICs had secured only 2.6 billion doses, with 1.1 billion of the total delivered through the COVAX facility.

Beyond the African context, system-level analyses from other regions highlight how structural, regulatory, and behavioral factors interact to influence vaccine implementation. For example, retrospective cohort studies and meta-analyses from Italy show that even where guidelines are clear, vulnerable groups such as splenectomised patients often have suboptimal coverage with recommended vaccines (66–68). This reveals gaps at the interface of policy design, service delivery, and patient education. Likewise, sero-epidemiological work on poliovirus neutralizing antibodies in polio-free settings demonstrates that waning immunity and uneven program performance can erode long-term gains if surveillance and catch-up strategies are not adequately resourced (69). Recent umbrella reviews and multi-country studies on vaccine hesitancy among health workers, students, and specific risk groups further highlight the role of social media exposure, trust dynamics, and organizational support in shaping vaccination behaviors (70, 71). Together, these studies suggest that the governance and regulatory challenges we identify in Africa must be addressed alongside behavioral and communication strategies if expanded manufacturing capacity is to translate into sustainable improvements in vaccine uptake.

While our rapid review focused primarily on the structural, legal, and investment frameworks shaping vaccine manufacturing capacity, these arrangements operate within socio-cultural environments that also influence vaccine production, distribution, and uptake in Africa. Empirical evidence suggests that corruption, perceptions of unfair vaccine allocation, and low institutional trust are closely linked to COVID-19 vaccine hesitancy and reduced uptake (72–75). Misinformation, which is circulated via social media, religious networks, and community rumors, alongside certain religious and cultural beliefs, has further undermined confidence in vaccines (76–78). Historical experiences of colonialism and biomedical exploitation, narratives that portray Africa as a “testing ground” for new products, continue to fuel suspicion towards COVID-19 vaccines and global health actors (79, 80). These socio-cultural dynamics interact with and sometimes blunt the effect of otherwise well-designed policy and legal frameworks (72–74). However, because our eligibility criteria and data extraction focused on policy, legal, governance, and investment instruments, we did not systematically capture evidence on socio-cultural determinants. Future reviews and empirical studies that explicitly integrate these dimensions would provide a more comprehensive understanding of the context in which vaccine R&D and manufacturing policies are implemented in Africa.

Aside from the inequitable distribution of manufacturing capacity, with Africa disproportionately burdened, global supply constraints on raw materials for these manufacturing companies pose a huge challenge. Africa's dependency on foreign suppliers has resulted in delays in the production of vaccines, drugs, and other medical supplies and products, as well as creating uncertainty in the vaccine production market and subsequently impacting the abilities of governments to implement vaccine rollout programmes and protect their population (17, 81). Export restrictions placed on vaccines, vaccine raw materials, and on the delivery of medical supplies such as syringes, glass vials, and stabilizing agents, have further undermined equity-focused partnerships geared towards building the capacities of African manufacturers (10). In addition, intellectual property rights, which have been reinforced by the TRIPS agreement, give patent holders exclusive rights to develop, market, and use the vaccines, vaccine raw materials, or other medical supplies for a period of 20 years (24). They exercise a monopoly over these supplies and grant access to partnering companies through voluntary licensing only to a small fraction of these resources. This limits access to the resources needed to scale up domestic production of vaccines and medicines.

While ACT-A and COVAX played important roles in mobilizing resources and delivering large volumes of doses to LMICs, our synthesis suggests they have been less successful in addressing the structural barriers highlighted in the Results section, particularly those related to local manufacturing capacity, technology transfer, and reliance on imported inputs. Similarly, the limited uptake of C-TAP by major manufacturers has meant that, in practice, the initiative has not substantially altered power asymmetries in access to vaccine-related IP and know-how for African producers. The adverse impact of the TRIPS agreement on access to vaccine raw materials, as well as its ability to impede the full implementation of COVAX and other economic, financial and trade agreements or frameworks in ensuring equitable access to vaccines and other medical products, emphasizes the importance of African governments taking frontline positions in decision making or agreements in international business or organizations or other agreements that are binding on them. The government needs to consider its context and the implications of such agreements for broader export, import, or trade arrangements before endorsing them.

To boost the capacity for innovation and vaccine development in Africa, African governments, manufacturing companies, and other key stakeholders need to strengthen these companies’ financial capacity by developing innovative ways to raise more money for vaccines. From the findings above, it is evident that most of the vaccine manufacturing companies in Africa rely heavily on external financial resources from developed countries and other international key players in the vaccine market (37). Although partnerships with these companies or key players enhance technical transfer, it is important for manufacturing companies to develop an effective strategy to raise sustainable investment capital. South Africa has made positive strides through its public-private partnerships, for instance, Biovac, for vaccine manufacturing (82). African governments and other key players in the manufacturing industry can map their private sector, focusing on capacities in the pharmaceutical industry or in institutions investing in research and development. It is additionally imperative for African countries to collaborate across the region in building their manufacturing capacities, as current reports on collaboration between sub-Saharan African countries are alarmingly low and pegged at 0.9% and 2.3% between researchers in West and Central Africa, and Southern Africa, respectively (62).

Emerging financing initiatives suggest a growing recognition that manufacturing sustainability depends not only on investment in production facilities but also on the creation of predictable markets for locally manufactured products. Mechanisms such as the African Vaccine Manufacturing Accelerator (AVMA), advance market commitments, and pooled procurement arrangements seek to address longstanding concerns regarding demand uncertainty and market fragmentation. Evidence from recent analyses suggests that demand creation and market-shaping interventions may be equally important as technology transfer and infrastructure investments in ensuring the long-term viability of African vaccine manufacturers.

Manufacturing companies can enter into public-private partnerships or product development partnerships with governments, private companies, organizations, or institutions shaping Africa's vaccine manufacturing industries, both within and across countries, to expand the research and development landscape for vaccine production. Manufacturing companies in Ghana, Nigeria, Kenya, Botswana, Uganda, and Rwanda who are now venturing into the vaccine manufacturing industry can enter into product development partnerships with South Africa's Biovac and Aspen or Tunisia's Institut Pasteur Tunis for the manufacturing of vaccines for measles, pneumococcal, tetanus, poliomyelitis, instead of having to invest in the entire process of making such vaccines.

Our recommendations are closely aligned with continental strategies already articulated by African institutions. The African Union's New Public Health Order emphasizes strengthening public health institutions and the workforce, expanding local manufacturing of vaccines, diagnostics, and therapeutics, increasing domestic investment in health security, and fostering action-oriented partnerships (83, 84). Africa CDC's Partnerships for African Vaccine Manufacturing (PAVM) further operationalizes this vision through a framework that aims to increase local production to 60% of Africa's vaccine needs by 2040 and to build an end-to-end vaccine R&D and manufacturing ecosystem (85–87). Similarly, the WHO mRNA technology transfer program, centered on a hub in South Africa and partner sites across Africa, demonstrates the potential of coordinated technology transfer, IP flexibilities, and regulatory strengthening to address some of the trade and IP barriers we highlight (88–90). Situating national reforms within these frameworks can help ensure that legal, policy, and financing changes at the country level contribute to a coherent continental agenda for vaccine equity and health sovereignty.

A recurring theme in recent studies is vaccine sovereignty, which extends beyond vaccine access to encompass the ability of countries and regions to develop, manufacture, procure, and distribute vaccines through resilient, sustainable systems. Closely related to the broader notion of pharmaceutical sovereignty, this perspective frames local vaccine manufacturing as a strategic component of health security, economic resilience, and preparedness for future pandemics (19, 20, 35). Several studies, therefore, advocate developing integrated vaccine manufacturing ecosystems that combine research and development, financing, technology transfer, regulatory strengthening, workforce development, and procurement systems within a coordinated continental framework (19, 20, 35).

Across the publications included in this review, several limitations of the underlying evidence base warrant attention. Many sources are grey literature, such as reports, policy briefs, and organizational websites, which often lack detailed methodological descriptions and may reflect institutional positions or advocacy goals. Even among peer-reviewed articles, most adopt descriptive or narrative designs rather than systematic evaluations of specific policy interventions. Few studies provide robust empirical estimates of the impact of particular frameworks or agreements on vaccine R&D capacity or manufacturing outputs. These limitations mean that the evidence base is stronger for mapping the existence and stated intentions of policies and initiatives than for assessing their effectiveness, and they underline the need for more rigorous, comparative, and longitudinal research on vaccine R&D and manufacturing policies in Africa. Finally, while our rapid review followed PRISMA guidance and employed a transparent screening and extraction process, the absence of a formal risk-of-bias assessment and the predominance of grey literature for some themes mean that our conclusions should be interpreted as mapping the policy and governance landscape rather than providing definitive causal estimates of impact.

## 5 Conclusion

This rapid review shows that Africa's aspiration to achieve vaccine equity and expand domestic vaccine manufacturing is underpinned by an emerging but still fragmented constellation of policy, legal, and governance frameworks. Across the literature and policy documents we reviewed, four cross-cutting challenges recur: dependence on imported vaccines and inputs; constrained use of intellectual property flexibilities and technology-transfer mechanisms; underinvestment in R&D and manufacturing infrastructure; and limited regulatory and governance capacity to coordinate regional initiatives.

At the same time, we identify important windows of opportunity. Continental strategies such as the African Union's New Public Health Order and Africa CDC's Partnerships for African Vaccine Manufacturing provide a coherent vision for expanding local production, strengthening regulatory systems, and increasing domestic investment by 2040. Initiatives such as the WHO mRNA technology transfer programme further illustrate how global solidarity mechanisms can support African manufacturers to move beyond fill-and-finish toward end-to-end production.

We recommend that African governments and regional bodies: (i) embed TRIPS flexibilities and pro-access principles into domestic legislation and regional trade agreements; (ii) expand predictable, long-term financing for vaccine R&D and manufacturing, including through innovative public–private and product development partnerships; (iii) invest in the institutional strengthening of national regulatory authorities and regional harmonisation efforts; and (iv) deliberately design stakeholder engagement models that incorporate civil society, academia and the private sector in agenda-setting and oversight. Implementing these measures in a coordinated way and aligning national reforms with continental priorities will be critical to translating Africa's latent capacity into sustainable and equitable access to vaccines for its populations and the wider global community.

## Data Availability

The original contributions presented in the study are included in the article/Supplementary Material, further inquiries can be directed to the corresponding author.
